# Supplementary material for: Distinct G protein-coupled receptor phosphorylation motifs modulate arrestin affinity and activation and global conformation
Source: Nat Commun. 2019 Mar 19;10:1261. doi: 10.1038/s41467-019-09204-y (PMC6424980; doi:10.1038/s41467-019-09204-y)
Supplement: Supplementary file 1 — Supplementary Information [file 41467_2019_9204_MOESM1_ESM.pdf]

Supplementary Information

Distinct G Protein-Coupled Receptor phosphorylation motifs modulate arrestin affinity and activation and global conformation

Mayer *et al.*

Supplementary Information

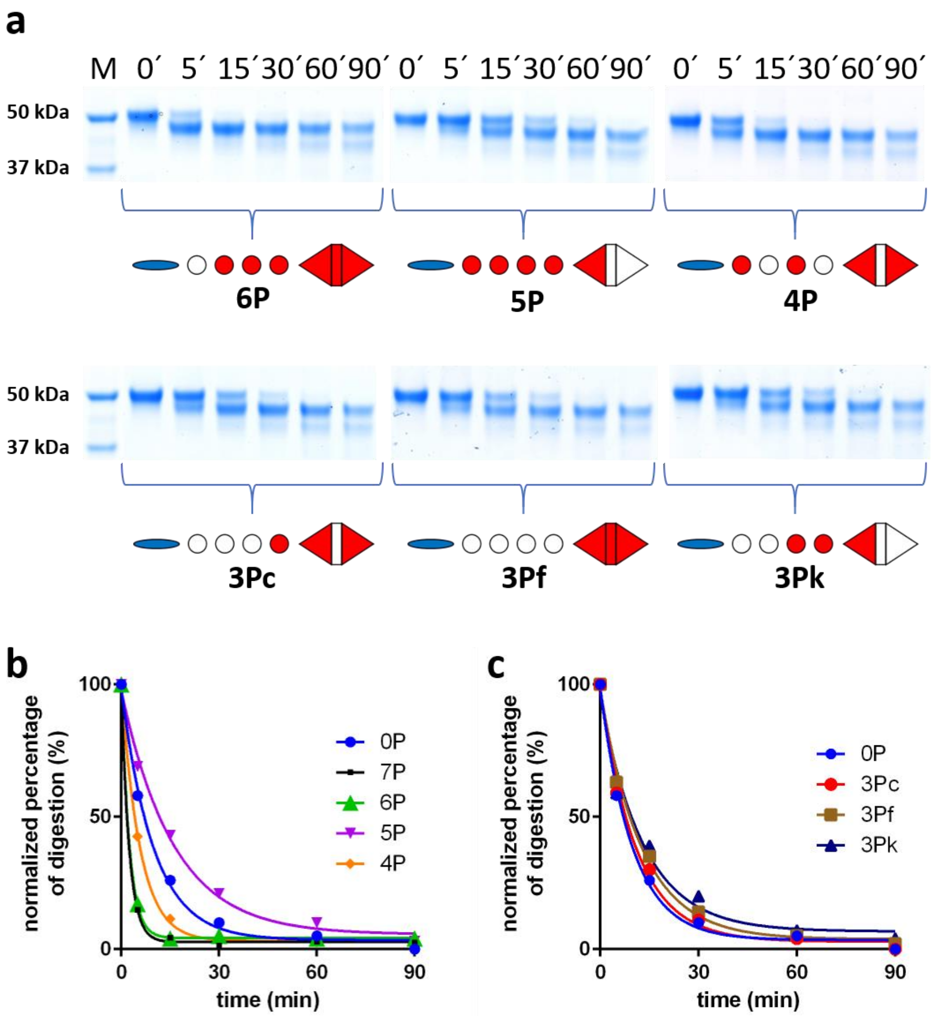

Supplementary Figure 1 Arrestin-2 digestion accelerates in presence of highly phosphorylated peptides with a notable exception

a) Limited trypsin digest of arrestin-2 in presence of representative phosphopeptide analogues of the rhodopsin C-terminus. The digestion of arrestin-2 always resulted in two bands. However, an acceleration in digestion was observed for more highly phosphorylated peptides such as 7P, 6P and 4P, but not for 5P. **b and c)** The percentage of digestion of the intact full-length arrestin-2 (uppermost band) was analyzed by ImageJ software and was plotted against the digestion time (n=1). The data was fitted by an exponential decay function in Prism6. The plot illustrates that the rate of digestion of intact arrestin-2 was increased in the presence of 7P, 6P (data overlays with the results of the 7P digestion) and 4P, which was not the case for other phosphorylated peptides or non-phosphorylated peptide 0P. In c, data for selected 3P peptides are shown which exhibit either strong- or non-binding behavior (Source data are provided as a Source Data file).

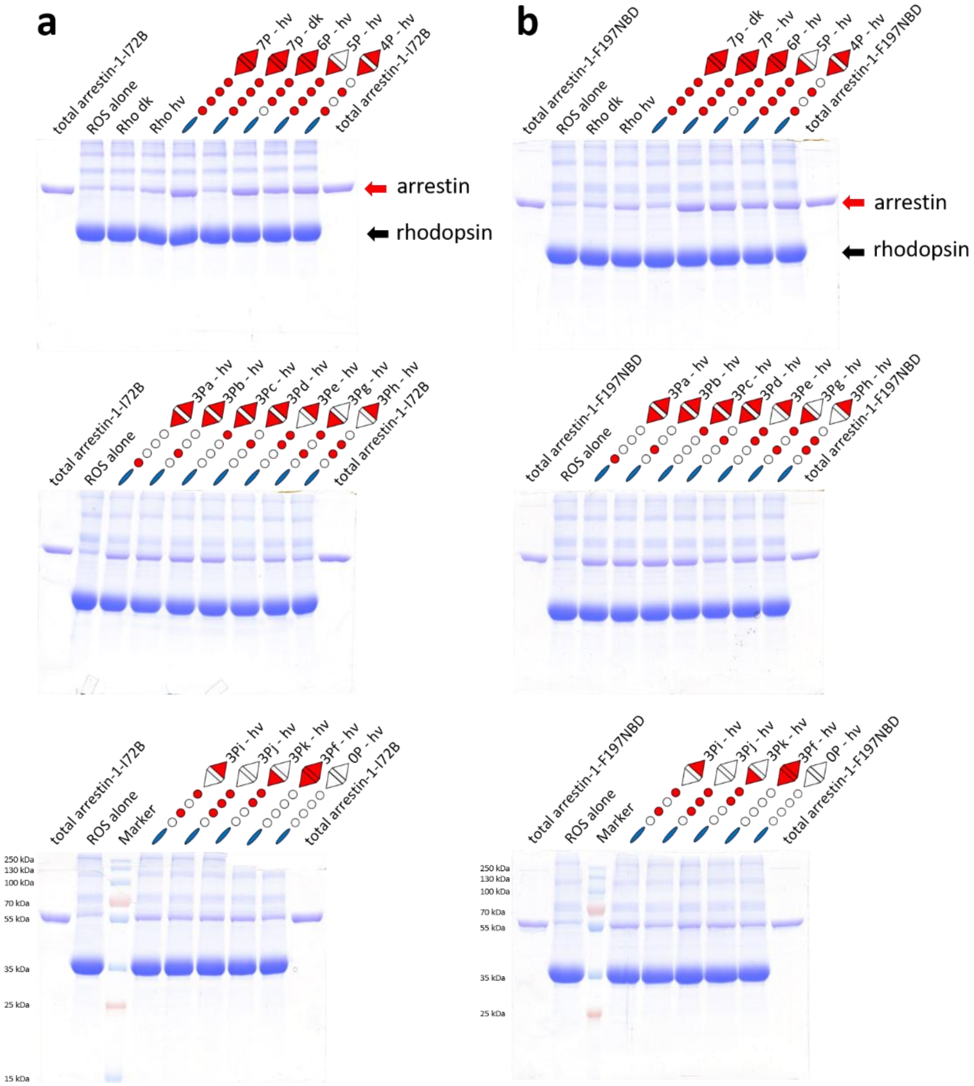

Supplementary Figure 2 Centrifugal pull down of fluorescently labelled mutants
Representative SDS-PAGE gels are shown for arrestin-1 mutants I72B (a) and F197NBD (b). Lane marked 'total arrestin' was loaded with the total amount of arrestin present in each pull-down experiment (1 μM, 2.25 μg). 'ROS alone' refers to a control containing only rhodopsin and no arrestin. 'Rho dk' refers to a pull-down performed with dark-state rhodopsin (no peptide), and 'Rho hυ' refers to pull-down performed with light-activated rhodopsin (no peptide). For arrestin-1 pull-down experiments performed with rhodopsin and phosphopeptide, the schematic of the peptide used (see Table 1) is placed above the lane. The arrows indicate the relative mobility of arrestin (red) and rhodopsin (black). Since only two protein species were present in these experiments, molecular weight marker was loaded onto only the bottom gels, for reference (Source data are provided as a Source Data file).

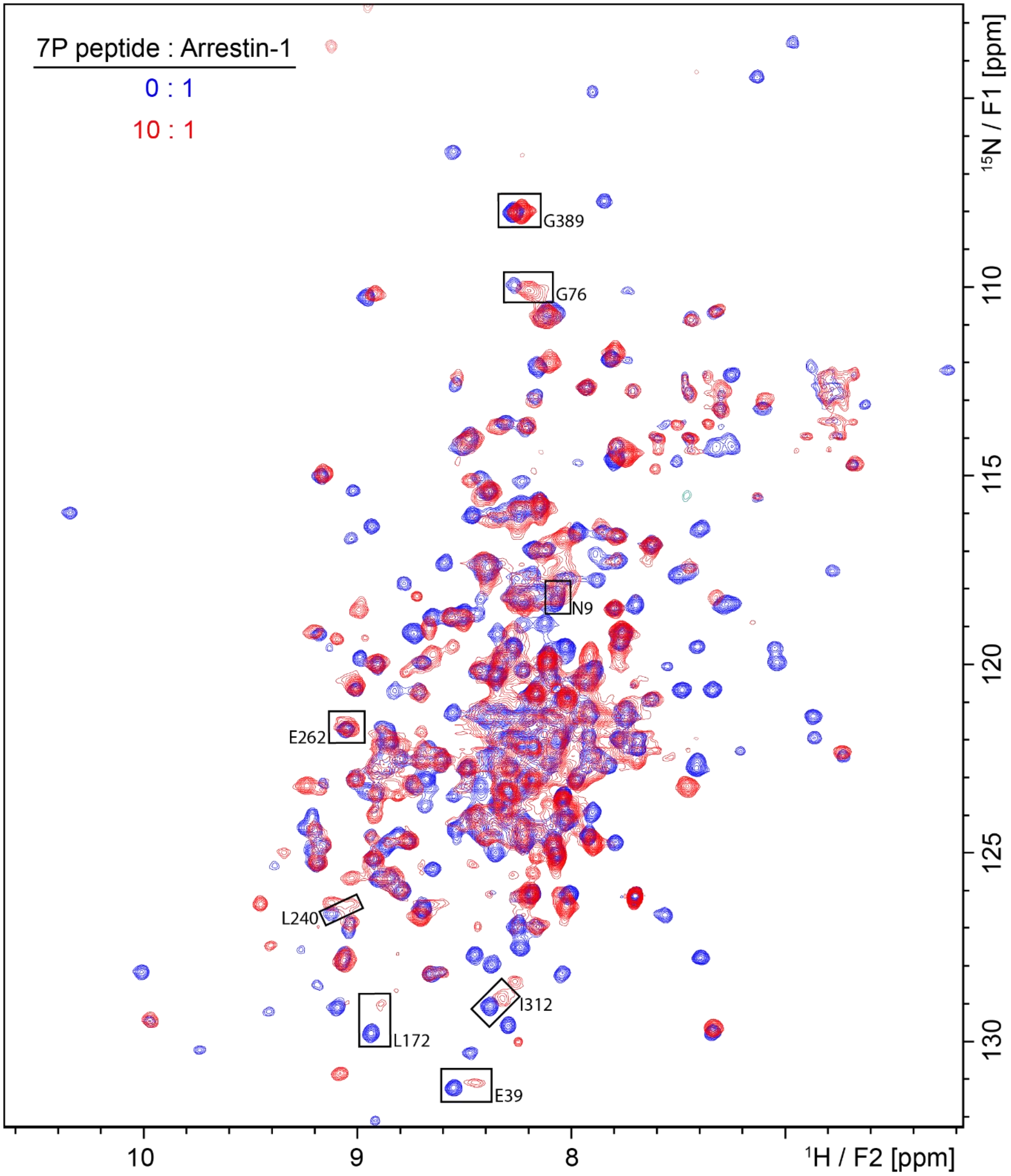

Supplementary Figure 3 NMR titration of arrestin-1 in presence of 7P
[^{15}N,^{1}H]TROSY spectra of ^{2}H,^{15}N,^{13}C-labelled arrestin-1 obtained before (blue) and after (red) addition of fully-phosphorylated peptide analogue of the bovine rhodopsin C-terminus (7P) at a stoichiometric ratio of 10 to 1 (1 mM phosphopeptide and 100 μM arrestin-1). Data were obtained at 600 MHz ^{1}H frequency. Positions of residues which were used to illustrate chemical shift changes of arrestin-1 upon adding the final peptide concentration in Figure 5 are indicated.

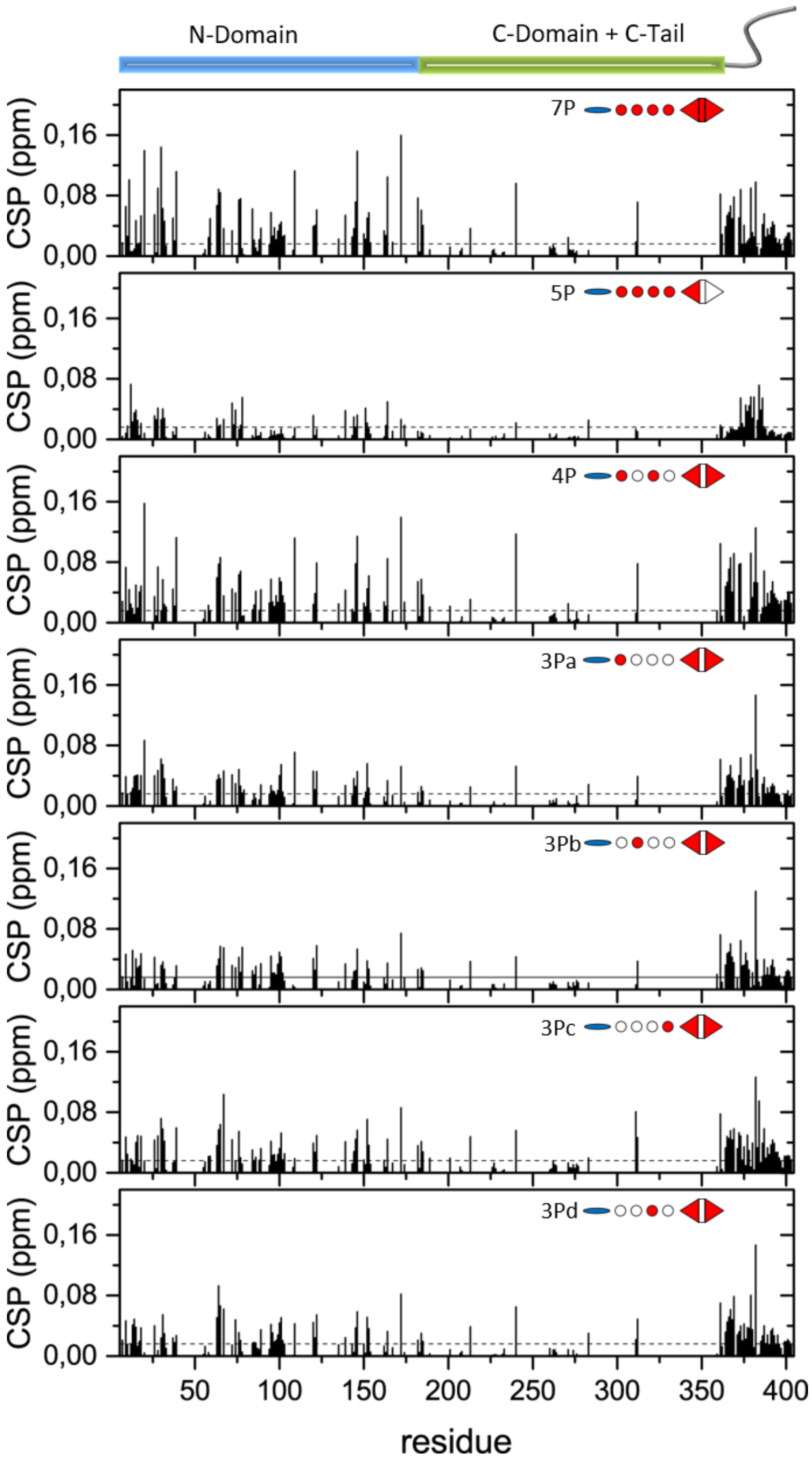

Supplementary Figure 4 CSP illustration of the individual NMR titrations

Barplots of the chemical shift perturbations (CSP) induced in arrestin-1 by differently phosphorylated peptides. In some cases, the TROSY signal disappeared before 10:1 peptide:Arrestin-1 ratio was reached. In this case the largest CSP which could be determined was used for the plot.

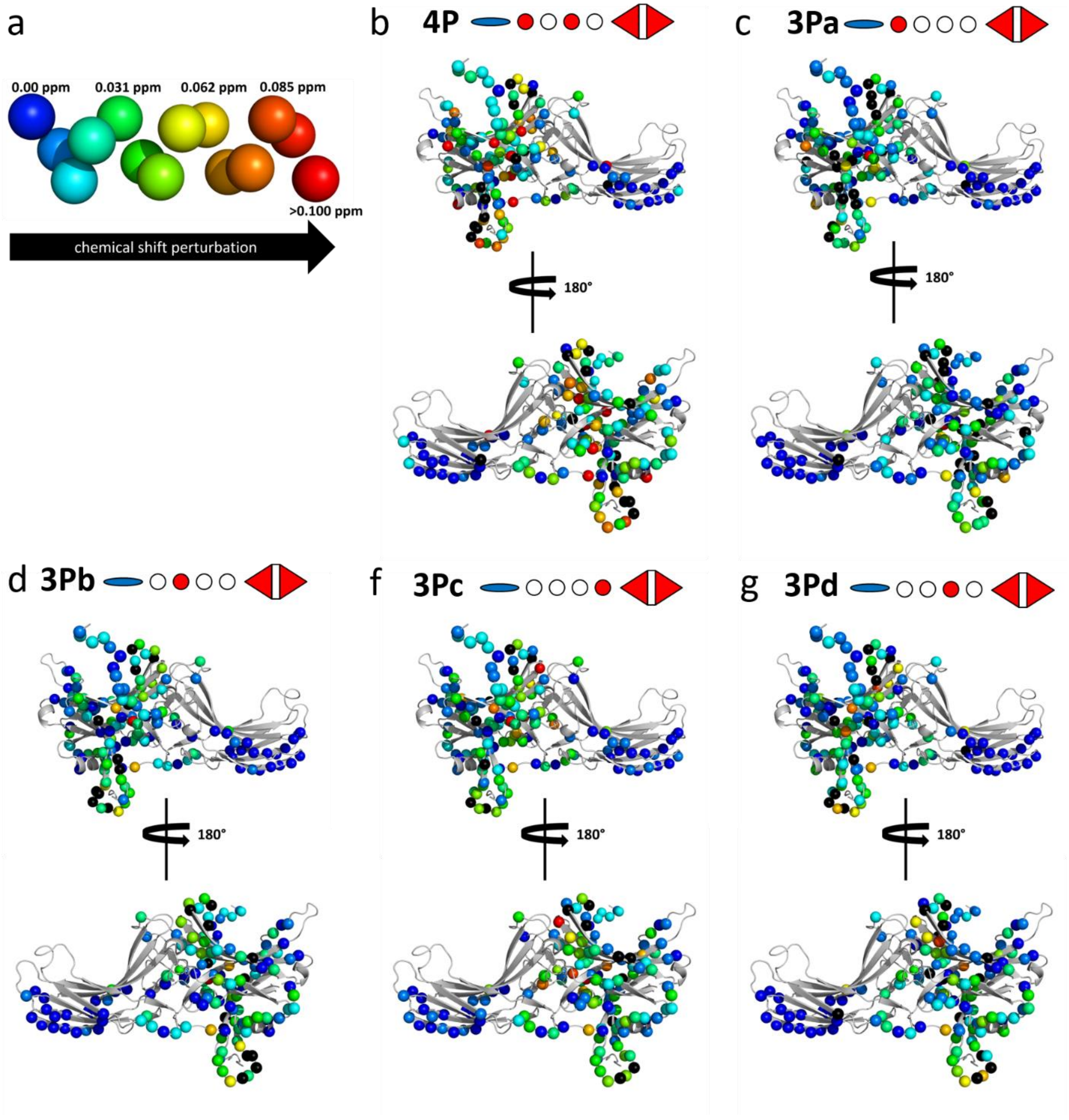

Supplementary Figure 5 Summary of CSP measured by NMR titration series

a) Color code indicating the magnitude of chemical shift perturbations. Residues which underwent significant line broadening during the titration so that their final position could not be determined are represented by black spheres. **b-h)** Chemical shift perturbations measured for different phosphopeptides, plotted on a structural model of basal arrestin-1 (PDB accession 1CF1, molecule D). The full length C-terminus, which is not resolved in the structure, was modelled as described in figure 6c.

**Supplementary Information Table S1.** Statistical analysis of Kd values for phosphopeptide binding, arrestin-1 versus arrestin-2 and arrestin-3																	
	Arr1																
	V2R PP	7P	6P	5P	4P	3Pa	3Pb	3Pc	3Pd	3Pe	3Pf	3Pg	3Ph	3Pi	3Pj	3Pk	0P
Arr 2	ns	ns	ns	****	ns	ns	ns	ns	ns	-	****	-	-	-	-	-	-
Arr 3	ns	ns	ns	ns	ns	****	****	****	****	-	ns	-	-	-	-	-	-

**Supplementary Information Table S2.** Statistical analysis of Kd values for phosphopeptide binding, arrestin-2 versus arrestin-3																	
	Arr2																
	V2R PP	7P	6P	5P	4P	3Pa	3Pb	3Pc	3Pd	3Pe	3Pf	3Pg	3Ph	3Pi	3Pj	3Pk	0P
Arr3	ns	ns	**	****	ns	****	****	****	****	-	****	-	-	-	-	-	-

**Supplementary Information Table S3.** Statistical analysis of Kd values for phosphopeptide binding by arrestin-1, arrestin-2 and arrestin-3, 7P vs. all other peptides																
	7P															
	V2R PP	6P	5P	4P	3Pa	3Pb	3Pc	3Pd	3Pe	3Pf	3Pg	3Ph	3Pi	3Pj	3Pk	0P
Arr1	ns	ns	****	ns	ns	*	ns	ns	ns	****	****	****	****	****	****	****
Arr2	ns	ns	ns	*	****	****	****	****	****	ns	****	****	****	****	****	****
Arr3	ns	ns	ns	ns	ns	****	****	**	**	****	****	****	****	****	****	****

Supplementary Information Tables 1, 2 and 3 Statistical analysis of the affinity measurements

The statistical analysis was performed by a standard ANOVA analysis in Prism6. 'ns', no statistical significance; *, P ≤ 0.05; **, P ≤ 0.01; ***, P ≤ 0.001; ****, P ≤ 0.0001; ' **-** ', no calculation was possible due to no measurable Kd value.

residue	7P /ppm	5P /ppm	4P /ppm	3Pa /ppm	3Pb /ppm	3Pc /ppm	3Pd /ppm
**A7**	0,018	0,004	0,028	0,018	0,017	0,017	0,021
**N9**	0,066	0,008	0,073	0,039	0,047	0,047	0,047
**H10**	0,026	0,018	0,015	0,016	0,010	0,025	0,016
**V11**	0,101	0,000	0,044	0,000	0,000	0,000	0,000
**I12**	0,006	0,073	0,025	0,018	0,015	0,018	0,016
**F13**	0,006	0,023	0,019	0,023	0,052	0,015	0,041
**K14**	0,009	0,035	0,011	0,039	0,005	0,010	0,049
**K15**	0,047	0,039	0,050	0,040	0,041	0,040	0,038
**I16**	0,016	0,025	0,019	0,041	0,029	0,049	0,013
**S17**	0,017	0,005	0,041	0,021	0,031	0,007	0,020
**R18**	0,053	0,021	0,049	0,040	0,047	0,048	0,037
**K20**	0,140	0,008	0,158	0,087	0,014	0,016	0,004
**L26**	0,055	0,031	0,035	0,040	0,043	0,043	0,040
**G27**	0,004	0,026	0,009	0,000	0,006	0,006	0,001
**K28**	0,090	0,041	0,074	0,047	0,008	0,049	0,007
**D30**	0,144	0,026	0,016	0,062	0,032	0,072	0,024
**Y31**	0,063	0,041	0,057	0,055	0,037	0,058	0,055
**I32**	0,046	0,028	0,024	0,036	0,027	0,042	0,030
**D33**	0,014	0,003	0,021	0,012	0,008	0,003	0,010
**R37**	0,050	0,010	0,045	0,036	0,006	0,013	0,024
**V38**	0,020	0,005	0,022	0,020	0,015	0,015	0,018
**E39**	0,112	0,016	0,113	0,026	0,031	0,059	0,027
**K55**	0,003	0,002	0,004	0,003	0,005	0,007	0,002
**R56**	0,009	0,010	0,016	0,013	0,010	0,015	0,013
**Y58**	0,025	0,006	0,023	0,001	0,007	0,021	0,005
**V59**	0,050	0,003	0,016	0,006	0,011	0,022	0,001
**C63**	0,067	0,028	0,060	0,034	0,031	0,036	0,051
**A64**	0,089	0,017	0,078	0,041	0,040	0,057	0,093
**F65**	0,084	0,019	0,086	0,036	0,057	0,064	0,066
**Y67**	0,036	0,026	0,036	0,046	0,055	0,104	0,062
**I72**	0,034	0,048	0,045	0,041	0,032	0,044	0,015
**D73**	0,000	0,019	0,000	0,000	0,000	0,000	0,000
**V74**	0,000	0,039	0,040	0,030	0,029	0,016	0,048
**M75**	0,000	0,000	0,000	0,000	0,000	0,000	0,000
**G76**	0,074	0,014	0,064	0,048	0,043	0,055	0,031
**L77**	0,076	0,019	0,068	0,027	0,023	0,020	0,021
**S78**	0,010	0,056	0,009	0,017	0,056	0,012	0,005
**F79**	0,002	0,000	0,009	0,021	0,000	0,000	0,000
**Y84**	0,062	0,005	0,017	0,007	0,025	0,030	0,017
**A85**	0,021	0,002	0,024	0,016	0,019	0,016	0,018
**S86**	0,011	0,014	0,042	0,010	0,031	0,021	0,017
**Q87**	0,006	0,004	0,000	0,000	0,000	0,000	0,010

residue	7P /ppm	5P /ppm	4P /ppm	3Pa /ppm	3Pb /ppm	3Pc /ppm	3Pd /ppm
V88	0,022	0,005	0,017	0,008	0,006	0,017	0,009
Q89	0,037	0,009	0,044	0,028	0,034	0,032	0,035
V94	0,015	0,003	0,026	0,016	0,003	0,010	0,009
G95	0,058	0,012	0,057	0,027	0,044	0,042	0,042
A96	0,026	0,005	0,027	0,020	0,022	0,023	0,031
S97	0,038	0,011	0,022	0,019	0,022	0,024	0,019
G98	0,022	0,005	0,036	0,015	0,019	0,015	0,021
A99	0,033	0,007	0,027	0,020	0,033	0,024	0,028
T100	0,042	0,007	0,059	0,040	0,049	0,032	0,044
T101	0,045	0,014	0,054	0,055	0,043	0,053	0,051
R102	0,026	0,007	0,036	0,019	0,022	0,017	0,020
L103	0,027	0,002	0,026	0,011	0,011	0,025	0,016
I108	0,008	0,004	0,001	0,003	0,005	0,007	0,001
K109	0,113	0,016	0,112	0,071	0,003	0,019	0,043
L120	0,039	0,032	0,024	0,046	0,041	0,039	0,045
T121	0,041	0,005	0,039	0,022	0,025	0,027	0,024
F122	0,061	0,014	0,079	0,046	0,058	0,049	0,055
A135	0,023	0,005	0,025	0,013	0,010	0,008	0,015
V139	0,054	0,038	0,043	0,027	0,034	0,041	0,025
C143	0,026	0,005	0,018	0,014	0,013	0,012	0,016
G144	0,036	0,030	0,014	0,036	0,024	0,028	0,005
V145	0,072	0,016	0,078	0,026	0,025	0,044	0,037
D146	0,139	0,032	0,114	0,046	0,054	0,056	0,059
K150	0,030	0,001	0,026	0,010	0,010	0,017	0,016
A151	0,022	0,041	0,021	0,018	0,007	0,011	0,004
F152	0,050	0,022	0,045	0,056	0,038	0,071	0,051
A153	0,057	0,014	0,062	0,024	0,027	0,037	0,036
T154	0,018	0,007	0,013	0,003	0,004	0,011	0,005
D162	0,034	0,003	0,028	0,012	0,015	0,017	0,014
K163	0,027	0,019	0,022	0,006	0,007	0,007	0,005
I164	0,105	0,050	0,085	0,034	0,035	0,044	0,033
K167	0,019	0,011	0,016	0,014	0,013	0,013	0,011
L172	0,160	0,027	0,139	0,052	0,074	0,086	0,082
I174	0,001	0,019	0,027	0,005	0,015	0,011	0,005
R182	0,077	0,011	0,054	0,018	0,026	0,036	0,021
D183	0,006	0,003	0,008	0,005	0,003	0,006	0,005
M184	0,061	0,010	0,058	0,026	0,029	0,041	0,030
G185	0,041	0,008	0,037	0,020	0,025	0,028	0,020
R189	0,009	0,005	0,021	0,008	0,001	0,019	0,003
K201	0,012	0,002	0,022	0,007	0,013	0,019	0,009
V207	0,006	0,001	0,002	0,003	0,004	0,008	0,004
S208	0,010	0,004	0,008	0,004	0,005	0,003	0,006

residue	7P /ppm	5P /ppm	4P /ppm	3Pa /ppm	3Pb /ppm	3Pc /ppm	3Pd /ppm
**I213**	0,037	0,013	0,031	0,025	0,037	0,048	0,039
**V226**	0,007	0,003	0,007	0,002	0,006	0,007	0,001
**T227**	0,009	0,000	0,006	0,006	0,006	0,013	0,001
**N228**	0,003	0,004	0,003	0,001	0,002	0,006	0,002
**K232**	0,005	0,002	0,004	0,000	0,001	0,003	0,002
**T233**	0,005	0,008	0,007	0,003	0,007	0,004	0,009
**L240**	0,096	0,022	0,117	0,052	0,043	0,056	0,065
**A260**	0,012	0,007	0,008	0,007	0,008	0,004	0,010
**A261**	0,010	0,001	0,009	0,003	0,007	0,005	0,000
**E262**	0,014	0,002	0,011	0,007	0,010	0,016	0,008
**E263**	0,011	0,003	0,013	0,004	0,007	0,011	0,008
**A264**	0,003	0,007	0,006	0,010	0,006	0,009	0,004
**N271**	0,025	0,002	0,025	0,006	0,010	0,013	0,009
**S272**	0,009	0,003	0,004	0,003	0,004	0,007	0,000
**S273**	0,007	0,001	0,001	0,001	0,002	0,006	0,000
**L274**	0,008	0,003	0,002	0,002	0,009	0,011	0,008
**T275**	0,004	0,004	0,003	0,003	0,004	0,006	0,000
**K276**	0,007	0,001	0,015	0,013	0,012	0,011	0,003
**T277**	0,000	0,003	0,005	0,003	0,009	0,009	0,000
**L283**	0,007	0,026	0,011	0,028	0,009	0,020	0,030
**I311**	0,019	0,014	0,013	0,008	0,006	0,081	0,022
**I312**	0,071	0,010	0,078	0,039	0,037	0,047	0,049
**Q359**	0,001	0,006	0,016	0,006	0,020	0,008	0,003
**E361**	0,082	0,019	0,105	0,062	0,072	0,078	0,070
**D362**	0,029	0,016	0,009	0,013	0,010	0,005	0,013
**D364**	0,039	0,003	0,048	0,024	0,032	0,033	0,032
**T365**	0,054	0,010	0,054	0,040	0,048	0,048	0,045
**A366**	0,058	0,008	0,071	0,041	0,050	0,045	0,051
**K367**	0,066	0,014	0,086	0,054	0,061	0,056	0,062
**E368**	0,050	0,014	0,041	0,037	0,043	0,044	0,048
**S369**	0,078	0,013	0,092	0,033	0,036	0,058	0,079
**F370**	0,000	0,012	0,000	0,000	0,000	0,000	0,000
**Q371**	0,000	0,013	0,000	0,023	0,015	0,032	0,028
**D372**	0,051	0,016	0,077	0,041	0,000	0,053	0,032
**E373**	0,088	0,055	0,078	0,064	0,065	0,049	0,040
**N374**	0,016	0,021	0,000	0,027	0,031	0,017	0,020
**F375**	0,041	0,016	0,008	0,034	0,032	0,034	0,043
**V376**	0,017	0,045	0,000	0,000	0,048	0,010	0,023
**F377**	0,020	0,037	0,000	0,000	0,038	0,050	0,038
**E378**	0,023	0,045	0,017	0,033	0,026	0,029	0,037
**E379**	0,090	0,056	0,092	0,068	0,002	0,008	0,080
**F380**	0,031	0,028	0,034	0,037	0,022	0,040	0,035

residue	7P /ppm	5P /ppm	4P /ppm	3Pa /ppm	3Pb /ppm	3Pc /ppm	3Pd /ppm
**A381**	0,025	0,056	0,053	0,000	0,005	0,004	0,011
**R382**	0,098	0,000	0,126	0,147	0,130	0,126	0,147
**Q383**	0,012	0,025	0,053	0,048	0,039	0,033	0,000
**N384**	0,000	0,072	0,000	0,013	0,010	0,095	0,002
**L385**	0,010	0,039	0,011	0,000	0,004	0,014	0,020
**K386**	0,042	0,055	0,020	0,029	0,018	0,039	0,027
**D387**	0,056	0,017	0,068	0,038	0,040	0,058	0,040
**A388**	0,025	0,014	0,021	0,015	0,019	0,020	0,017
**G389**	0,036	0,005	0,039	0,024	0,029	0,033	0,028
**E390**	0,028	0,009	0,026	0,017	0,019	0,024	0,015
**Y391**	0,038	0,011	0,042	0,025	0,031	0,037	0,033
**K392**	0,045	0,012	0,054	0,029	0,039	0,043	0,036
**E393**	0,041	0,009	0,038	0,026	0,029	0,039	0,027
**E394**	0,024	0,003	0,032	0,016	0,017	0,027	0,015
**K395**	0,017	0,006	0,030	0,017	0,017	0,027	0,028
**T396**	0,022	0,006	0,024	0,014	0,018	0,020	0,016
**D397**	0,015	0,008	0,029	0,005	0,009	0,016	0,010
**Q398**	0,007	0,001	0,000	0,000	0,005	0,006	0,002
**E399**	0,023	0,005	0,030	0,015	0,017	0,022	0,019
**A400**	0,026	0,007	0,030	0,016	0,023	0,022	0,019
**A401**	0,030	0,009	0,039	0,020	0,026	0,023	0,022
**M402**	0,031	0,009	0,037	0,013	0,024	0,022	0,019
**D403**	0,021	0,009	0,026	0,015	0,016	0,017	0,016

Supplementary Information Table 4 Chemical shift perturbations in arrestin-1

Chemical shift perturbations of all assigned residues of arrestin-1 observed in [^{15}N,^{1}H]TROSY NMR upon titrating seven phosphopeptides up to a ratio of 10:1 peptide:arrestin-1. In some cases, the TROSY signal disappeared before 10:1 peptide:Arrestin-1 ratio was reached. In this case the largest CSP which could be determined is listed.

	Primer Sequence
ForwardVector	ATGCTCGAGGATCCGGCTG
ReverseVector	GGATCCACCCTGGAAGTACAGG
Forward_Arrestin-1 wilde-type and mutantF85AF197A	ACTTCCAGGGTGGATCCGCAAACAAACCCGCACCAAATC
Reverse_Arrestin-1 wilde-type and mutantF85AF197A	CGGATCCTCGAGCATTTATTCGTCCATTGCCGCTTCCTG
Forward_Arrestin-2 wilde-type	CTTCCAGGGTGGATCCGGTGATAAAGGCACCCGCG
Reverse_Arrestin-2 wilde-type	CGGATCCTCGAGCATTTAGCCGCTACCGCGATTATTCA
Forward_Arrestin-3 wilde-type	CTTCCAGGGTGGATCCGGTGAAAAGCCTGGCACCC
Reverse_Arrestin-3 wilde-type	CGGATCCTCGAGCATTTAACACAGCTGGTCGTCATAGTCG

Supplementary Information Table 5 Primer design for PCR

The primers are design for the Gibson Assembly protocol. The vector primers are the same for all four constructs.

Supplementary Note 1

Two exceptions are the ADRB2 and ADA1B receptors, which appear to have a negatively charged region. One potential reason for the class A-like behavior of these receptors is that these sites are not phosphorylated in cells. Another cause could be that the negatively charged motif is too far from the receptor core to sufficiently activate/recruit arrestin for receptor binding. In general, the Class B GPCRs have shorter C-termini, which may be important for forming stable complexes with arrestins. Besides increasing the distance between phosphorylation sites and the receptor core, a longer C-terminus might increase exposure of all phosphorylation sites to phosphatases, leading to dephosphorylation and decreasing the likelihood that both motifs are phosphorylated at the same time.
