# Supplementary material for: Distinct G protein-coupled receptor phosphorylation motifs modulate arrestin affinity and activation and global conformation
Source: Nat Commun. 2019 Mar 19;10:1261. doi: 10.1038/s41467-019-09204-y (PMC6424980; doi:10.1038/s41467-019-09204-y)
Supplement: Supplementary file 3 — Reporting Summary [file 41467_2019_9204_MOESM3_ESM.pdf]

natureresearch

Corresponding author(s): Daniel Mayer
Dmitry B. Veprintsev

Reporting Summary

Nature Research wishes to improve the reproducibility of the work that we publish. This form provides structure for consistency and transparency in reporting. For further information on Nature Research policies, see Authors & Referees and the Editorial Policy Checklist.

Statistical parameters

When statistical analyses are reported, confirm that the following items are present in the relevant location (e.g. figure legend, table legend, main text, or Methods section).

n/a	Confirmed	
☐	☒	The exact sample size (n) for each experimental group/condition, given as a discrete number and unit of measurement
☐	☒	An indication of whether measurements were taken from distinct samples or whether the same sample was measured repeatedly
☒	☐	The statistical test(s) used AND whether they are one- or two-sided<br>*Only common tests should be described solely by name; describe more complex techniques in the Methods section.*
☒	☐	A description of all covariates tested
☒	☐	A description of any assumptions or corrections, such as tests of normality and adjustment for multiple comparisons
☒	☐	A full description of the statistics including central tendency (e.g. means) or other basic estimates (e.g. regression coefficient) AND variation (e.g. standard deviation) or associated estimates of uncertainty (e.g. confidence intervals)
☒	☐	For null hypothesis testing, the test statistic (e.g. F, t, r) with confidence intervals, effect sizes, degrees of freedom and P value noted<br>*Give P values as exact values whenever suitable.*
☒	☐	For Bayesian analysis, information on the choice of priors and Markov chain Monte Carlo settings
☒	☐	For hierarchical and complex designs, identification of the appropriate level for tests and full reporting of outcomes
☒	☐	Estimates of effect sizes (e.g. Cohen's d, Pearson's r), indicating how they were calculated
☐	☒	Clearly defined error bars<br>*State explicitly what error bars represent (e.g. SD, SE, CI)*

Our web collection on statistics for biologists may be useful.

Software and code

Policy information about availability of computer code

Data collection	PERAstar, Topspin; SPEX Fluorolog
Data analysis	Origin2016, CARA, Microsoft Excel, R, PyMOL, Prism6

For manuscripts utilizing custom algorithms or software that are central to the research but not yet described in published literature, software must be made available to editors/reviewers upon request. We strongly encourage code deposition in a community repository (e.g. GitHub). See the Nature Research guidelines for submitting code & software for further information.

Data

Policy information about availability of data

All manuscripts must include a data availability statement. This statement should provide the following information, where applicable:

- Accession codes, unique identifiers, or web links for publicly available datasets
- A list of figures that have associated raw data
- A description of any restrictions on data availability

Raw data available upon request.

Field-specific reporting

Please select the best fit for your research. If you are not sure, read the appropriate sections before making your selection.

☒ Life sciences ☐ Behavioural & social sciences ☐ Ecological, evolutionary & environmental sciences

For a reference copy of the document with all sections, see nature.com/authors/policies/ReportingSummary-flat.pdf

Life sciences study design

All studies must disclose on these points even when the disclosure is negative.

Sample size	The exact sample size (n) for each experimental group/condition, given as a discrete number and unit of measurement
Data exclusions	No data was excluded from the analysis.
Replication	For the peptide array, the experiments were taken across the whole array simultaneously. The array contained in total 384 spots in duplicates (384x2 individual data points). Each peptide was independently synthesized twice and printed twice on the array surface. The data presented and analyzed in the manuscript were measured on three reprints of the array, although several peptide arrays were used for optimizing the optimal condition for buffer composition and incubation time.<br>NMR spectra were recorded once per condition, except for the APO which was used as control for each individual titration series to ensure to have the exact same buffer condition and instrument settings. Datapoints were averaged for the individual titration steps over a time-frame of two days where we didn't observe changes in sample conditions.<br>Fluorescence anisotropy measurements were preformed for each peptide in triplicate.<br>Trypsin digest assay were performed for arrestin-1 in triplicate to ensure the reproducibility of the assay. No, variation in the final digestion pattern were observed between the individual repeats. Therefore, the digestion of arrestin-2 and -3 was done only once for each peptide.<br>Site directed fluorescence spectroscopy experiment (n=3-5) and the corresponding pull-down experiments were performed mostly in triplicate with a few expection where only two repeats were performed.
Randomization	No randomization was performed.
Blinding	No blinding was applied.

Reporting for specific materials, systems and methods

Materials & experimental systems

n/a	Involved in the study	
☒	☐	Unique biological materials
☒	☐	Antibodies
☒	☐	Eukaryotic cell lines
☒	☐	Palaeontology
☒	☐	Animals and other organisms
☒	☐	Human research participants

Methods

n/a	Involved in the study	
☒	☐	ChIP-seq
☒	☐	Flow cytometry
☒	☐	MRI-based neuroimaging
